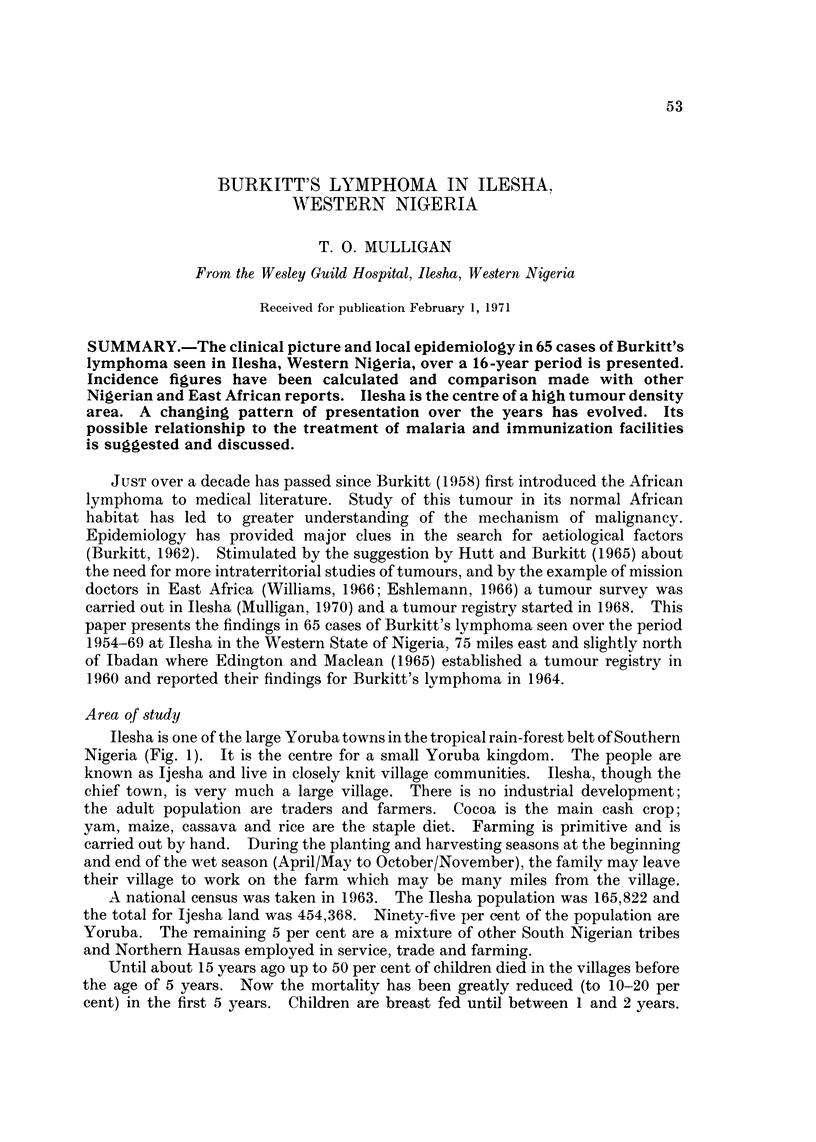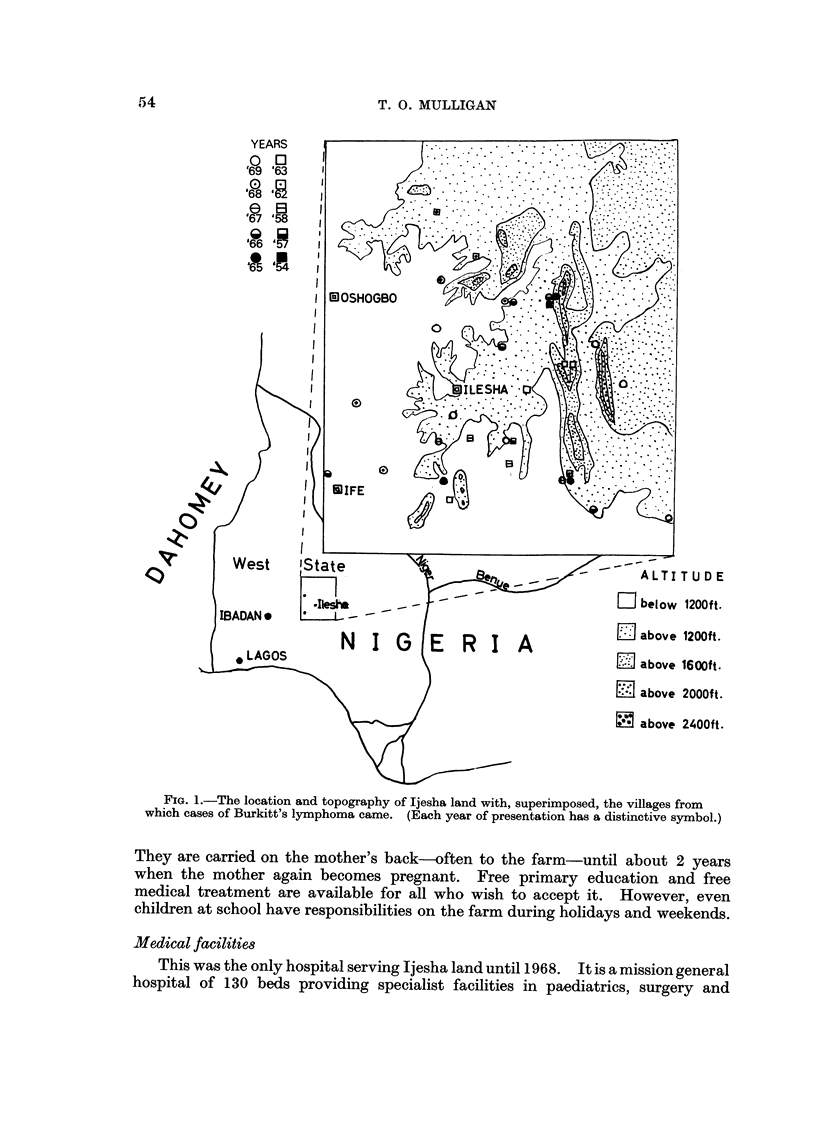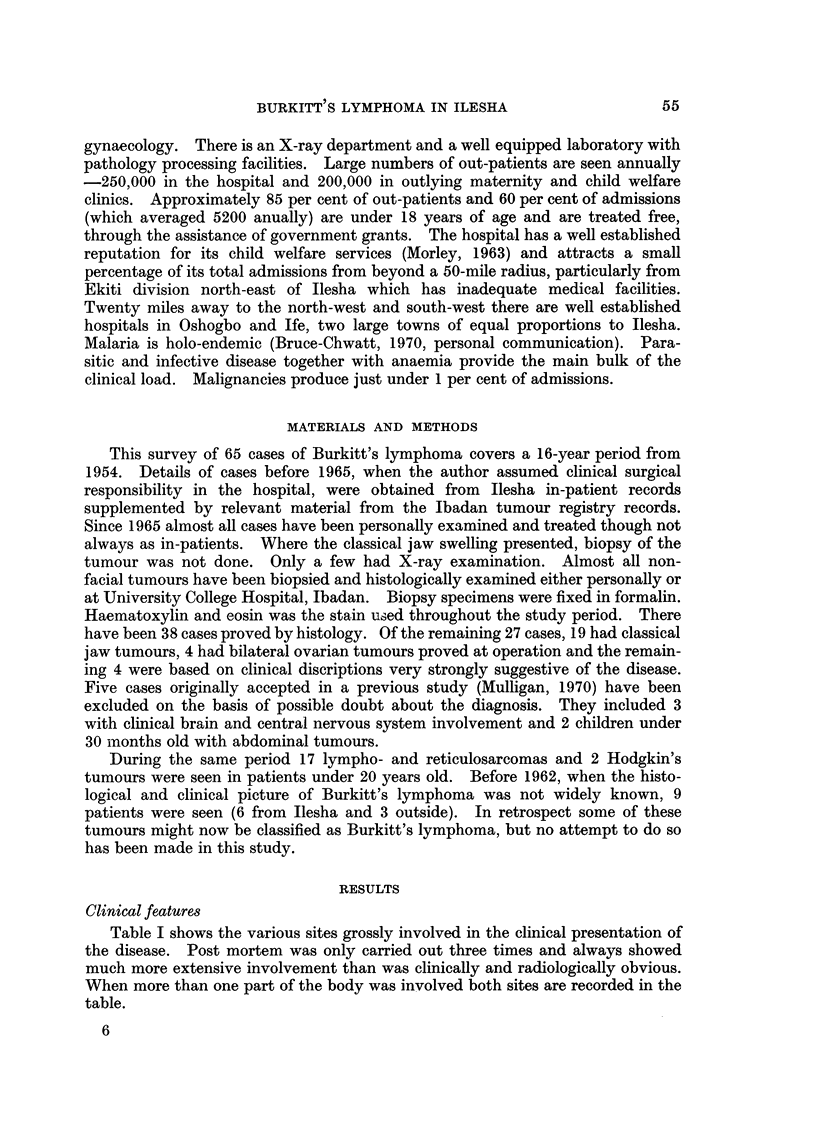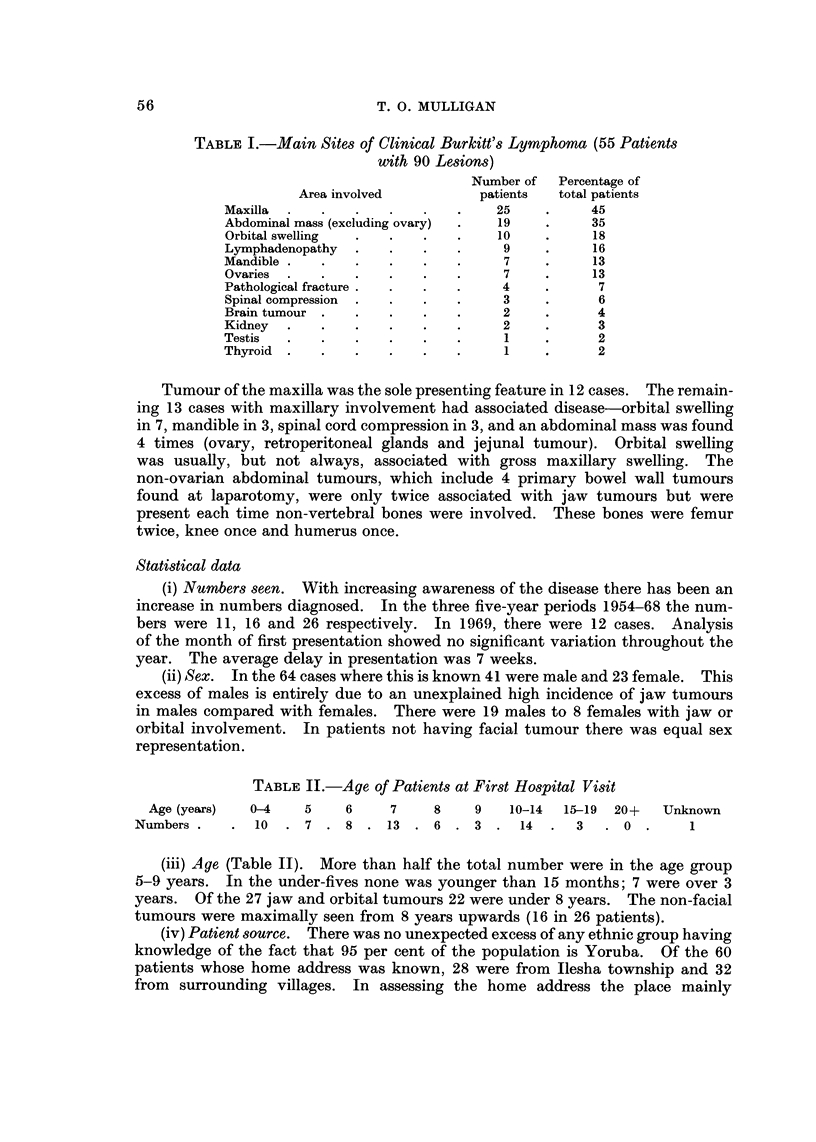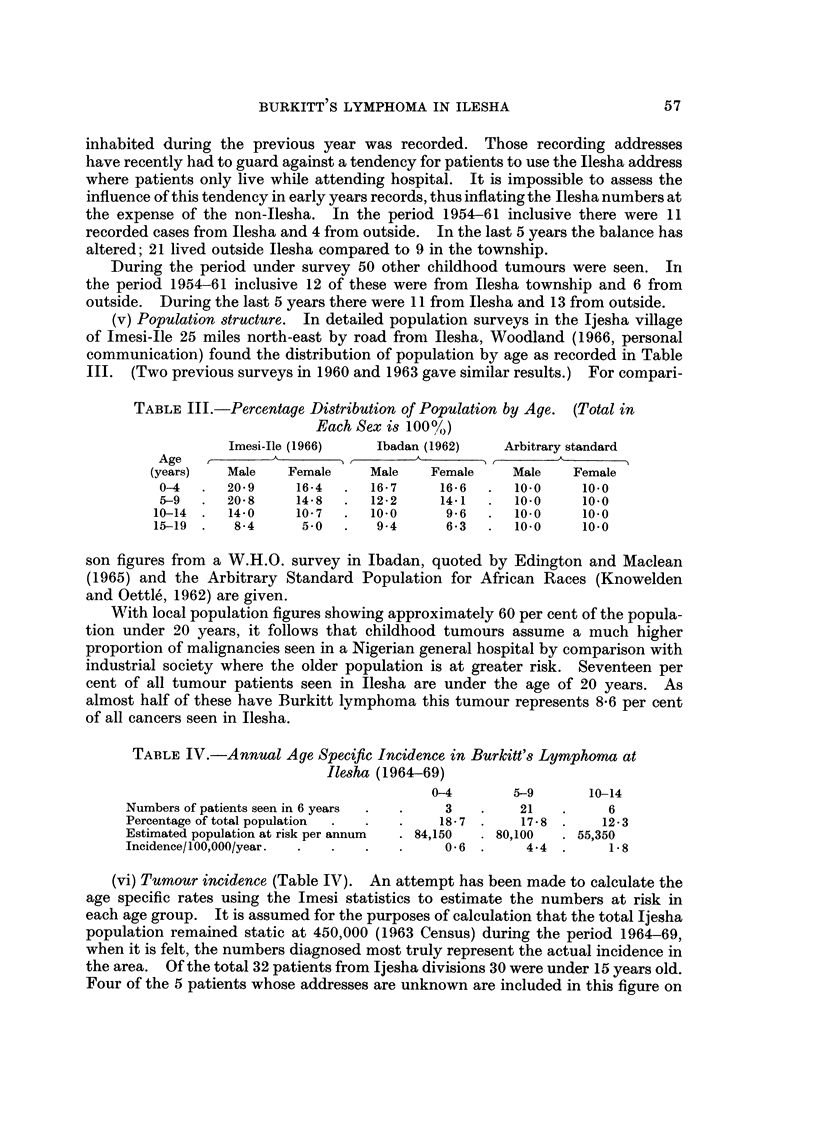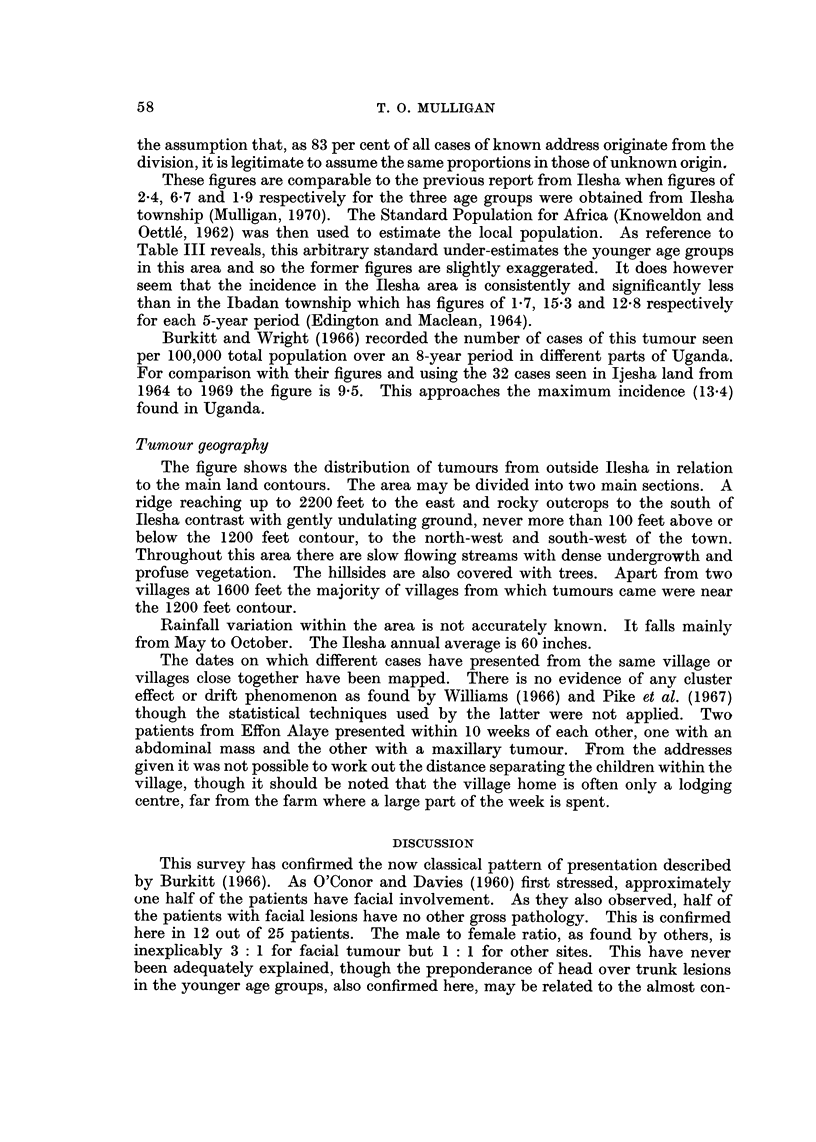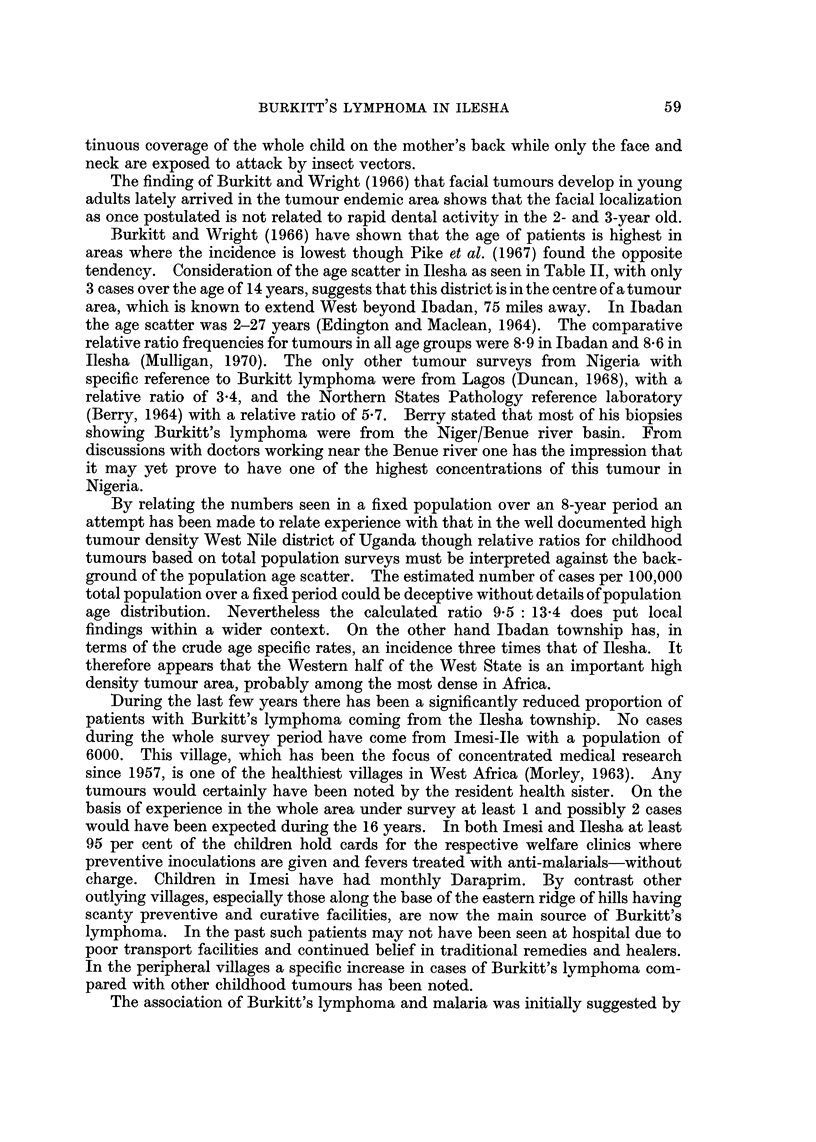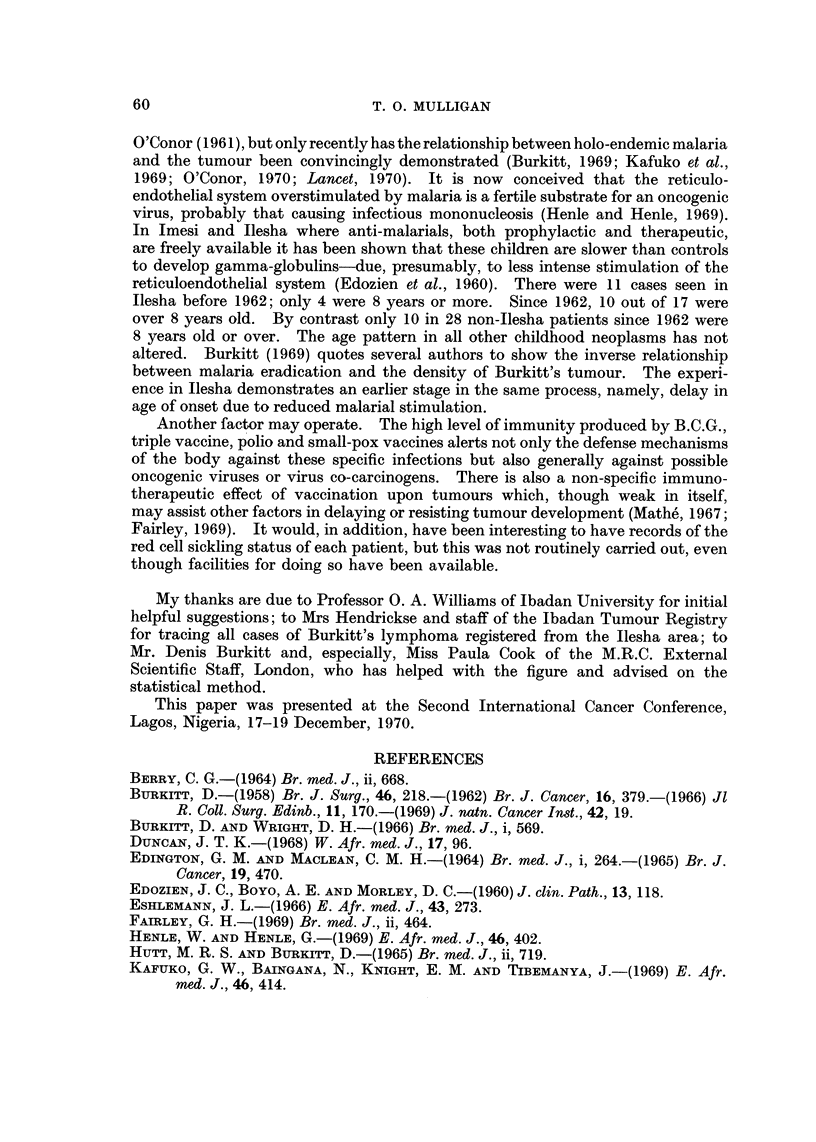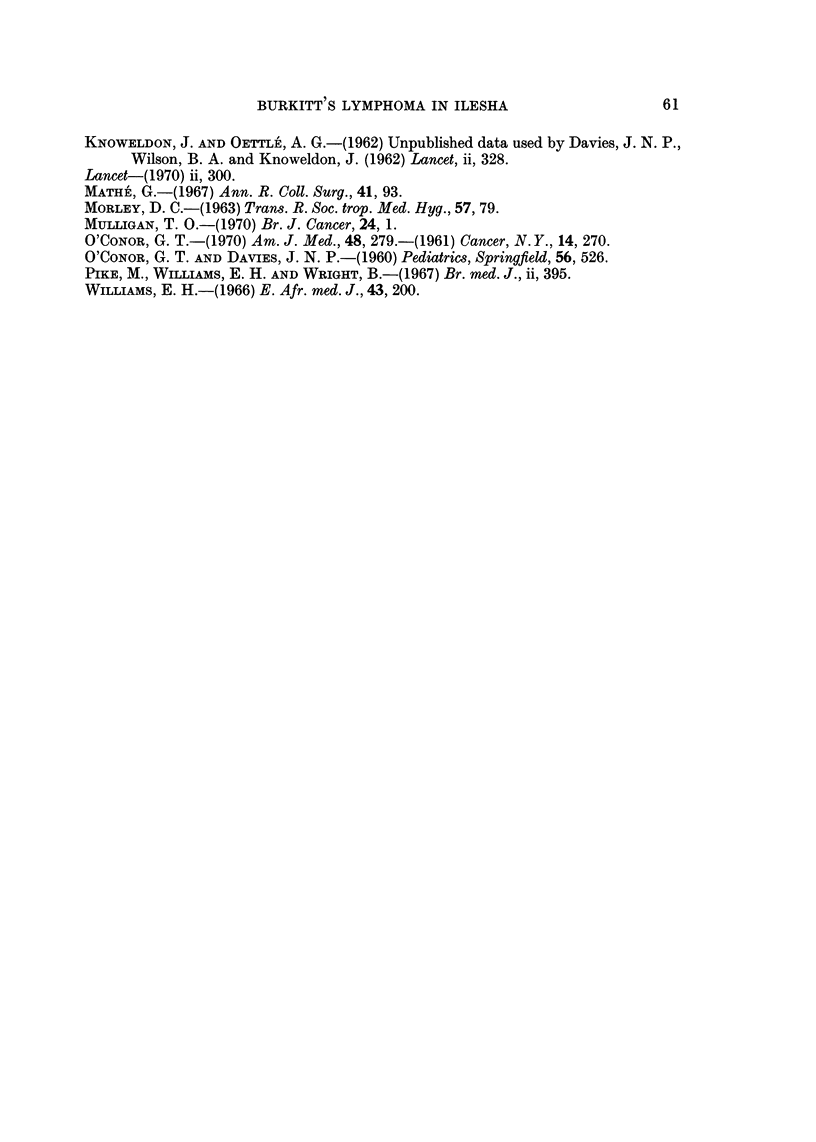# Burkitt's Lymphoma in Ilesha, Western Nigeria

**DOI:** 10.1038/bjc.1971.8

**Published:** 1971-03

**Authors:** T. O. Mulligan

## Abstract

The clinical picture and local epidemiology in 65 cases of Burkitt's lymphoma seen in Ilesha, Western Nigeria, over a 16-year period is presented. Incidence figures have been calculated and comparison made with other Nigerian and East African reports. Ilesha is the centre of a high tumour density area. A changing pattern of presentation over the years has evolved. Its possible relationship to the treatment of malaria and immunization facilities is suggested and discussed.


### BURKITT'S LYMPHOMA IN ILESHA, WESTERN NIGERIA

T. O. MULLIGAN

*From the Wesley Guild Hospital, Ilesha, Western Nigeria*

Received for publication February 1, 1971

**SUMMARY.—The clinical picture and local epidemiology in 65 cases of Burkitt's lymphoma seen in Ilesha, Western Nigeria, over a 16-year period is presented. Incidence figures have been calculated and comparison made with other Nigerian and East African reports. Ilesha is the centre of a high tumour density area. A changing pattern of presentation over the years has evolved. Its possible relationship to the treatment of malaria and immunization facilities is suggested and discussed.**

JUST over a decade has passed since Burkitt (1958) first introduced the African lymphoma to medical literature. Study of this tumour in its normal African habitat has led to greater understanding of the mechanism of malignancy. Epidemiology has provided major clues in the search for aetiological factors (Burkitt, 1962). Stimulated by the suggestion by Hutt and Burkitt (1965) about the need for more intraterritorial studies of tumours, and by the example of mission doctors in East Africa (Williams, 1966; Eshlemann, 1966) a tumour survey was carried out in Ilesha (Mulligan, 1970) and a tumour registry started in 1968. This paper presents the findings in 65 cases of Burkitt's lymphoma seen over the period 1954–69 at Ilesha in the Western State of Nigeria, 75 miles east and slightly north of Ibadan where Edington and Maclean (1965) established a tumour registry in 1960 and reported their findings for Burkitt's lymphoma in 1964.

*Area of study*

Ilesha is one of the large Yoruba towns in the tropical rain-forest belt of Southern Nigeria (Fig. 1). It is the centre for a small Yoruba kingdom. The people are known as Ijesha and live in closely knit village communities. Ilesha, though the chief town, is very much a large village. There is no industrial development; the adult population are traders and farmers. Cocoa is the main cash crop; yam, maize, cassava and rice are the staple diet. Farming is primitive and is carried out by hand. During the planting and harvesting seasons at the beginning and end of the wet season (April/May to October/November), the family may leave their village to work on the farm which may be many miles from the village.

A national census was taken in 1963. The Ilesha population was 165,822 and the total for Ijesha land was 454,368. Ninety-five per cent of the population are Yoruba. The remaining 5 per cent are a mixture of other South Nigerian tribes and Northern Hausas employed in service, trade and farming.

Until about 15 years ago up to 50 per cent of children died in the villages before the age of 5 years. Now the mortality has been greatly reduced (to 10–20 per cent) in the first 5 years. Children are breast fed until between 1 and 2 years.

FIG. 1.—The location and topography of Ijesha land with, superimposed, the villages from which cases of Burkitt's lymphoma came. (Each year of presentation has a distinctive symbol.)

They are carried on the mother's back—often to the farm—until about 2 years when the mother again becomes pregnant. Free primary education and free medical treatment are available for all who wish to accept it. However, even children at school have responsibilities on the farm during holidays and weekends.

*Medical facilities*

This was the only hospital serving Ijesha land until 1968. It is a mission general hospital of 130 beds providing specialist facilities in paediatrics, surgery and gynaecology. There is an X-ray department and a well equipped laboratory with pathology processing facilities. Large numbers of out-patients are seen annually —250,000 in the hospital and 200,000 in outlying maternity and child welfare clinics. Approximately 85 per cent of out-patients and 60 per cent of admissions (which averaged 5200 anually) are under 18 years of age and are treated free, through the assistance of government grants. The hospital has a well established reputation for its child welfare services (Morley, 1963) and attracts a small percentage of its total admissions from beyond a 50-mile radius, particularly from Ekiti division north-east of Ilesha which has inadequate medical facilities. Twenty miles away to the north-west and south-west there are well established hospitals in Oshogbo and Ife, two large towns of equal proportions to Ilesha. Malaria is holo-endemic (Bruce-Chwatt, 1970, personal communication). Parasitic and infective disease together with anaemia provide the main bulk of the clinical load. Malignancies produce just under 1 per cent of admissions.

### MATERIALS AND METHODS

This survey of 65 cases of Burkitt's lymphoma covers a 16-year period from 1954. Details of cases before 1965, when the author assumed clinical surgical responsibility in the hospital, were obtained from Ilesha in-patient records supplemented by relevant material from the Ibadan tumour registry records. Since 1965 almost all cases have been personally examined and treated though not always as in-patients. Where the classical jaw swelling presented, biopsy of the tumour was not done. Only a few had X-ray examination. Almost all non-facial tumours have been biopsied and histologically examined either personally or at University College Hospital, Ibadan. Biopsy specimens were fixed in formalin. Haematoxylin and eosin was the stain used throughout the study period. There have been 38 cases proved by histology. Of the remaining 27 cases, 19 had classical jaw tumours, 4 had bilateral ovarian tumours proved at operation and the remaining 4 were based on clinical discriptions very strongly suggestive of the disease. Five cases originally accepted in a previous study (Mulligan, 1970) have been excluded on the basis of possible doubt about the diagnosis. They included 3 with clinical brain and central nervous system involvement and 2 children under 30 months old with abdominal tumours.

During the same period 17 lympho- and reticulosarcomas and 2 Hodgkin's tumours were seen in patients under 20 years old. Before 1962, when the histological and clinical picture of Burkitt's lymphoma was not widely known, 9 patients were seen (6 from Ilesha and 3 outside). In retrospect some of these tumours might now be classified as Burkitt's lymphoma, but no attempt to do so has been made in this study.

### RESULTS

### *Clinical features*

Table I shows the various sites grossly involved in the clinical presentation of the disease. Post mortem was only carried out three times and always showed much more extensive involvement than was clinically and radiologically obvious. When more than one part of the body was involved both sites are recorded in the table.

TABLE I.—*Main Sites of Clinical Burkitt's Lymphoma (55 Patients with 90 Lesions)*

| Area involved | Number of patients | Percentage of total patients |
|---|---|---|
| Maxilla | 25 | 45 |
| Abdominal mass (excluding ovary) | 19 | 35 |
| Orbital swelling | 10 | 18 |
| Lymphadenopathy | 9 | 16 |
| Mandible | 7 | 13 |
| Ovaries | 7 | 13 |
| Pathological fracture | 4 | 7 |
| Spinal compression | 3 | 6 |
| Brain tumour | 2 | 4 |
| Kidney | 2 | 3 |
| Testis | 1 | 2 |
| Thyroid | 1 | 2 |

Tumour of the maxilla was the sole presenting feature in 12 cases. The remaining 13 cases with maxillary involvement had associated disease—orbital swelling in 7, mandible in 3, spinal cord compression in 3, and an abdominal mass was found 4 times (ovary, retroperitoneal glands and jejunal tumour). Orbital swelling was usually, but not always, associated with gross maxillary swelling. The non-ovarian abdominal tumours, which include 4 primary bowel wall tumours found at laparotomy, were only twice associated with jaw tumours but were present each time non-vertebral bones were involved. These bones were femur twice, knee once and humerus once.

*Statistical data*

(i) *Numbers seen.* With increasing awareness of the disease there has been an increase in numbers diagnosed. In the three five-year periods 1954–68 the numbers were 11, 16 and 26 respectively. In 1969, there were 12 cases. Analysis of the month of first presentation showed no significant variation throughout the year. The average delay in presentation was 7 weeks.

(ii) *Sex.* In the 64 cases where this is known 41 were male and 23 female. This excess of males is entirely due to an unexplained high incidence of jaw tumours in males compared with females. There were 19 males to 8 females with jaw or orbital involvement. In patients not having facial tumour there was equal sex representation.

TABLE II.—*Age of Patients at First Hospital Visit*

| Age (years) | 0–4 | 5 | 6 | 7 | 8 | 9 | 10–14 | 15–19 | 20+ | Unknown |
|---|---|---|---|---|---|---|---|---|---|---|
| Numbers | 10 | 7 | 8 | 13 | 6 | 3 | 14 | 3 | 0 | 1 |

(iii) *Age* (Table II). More than half the total number were in the age group 5–9 years. In the under-fives none was younger than 15 months; 7 were over 3 years. Of the 27 jaw and orbital tumours 22 were under 8 years. The non-facial tumours were maximally seen from 8 years upwards (16 in 26 patients).

(iv) *Patient source.* There was no unexpected excess of any ethnic group having knowledge of the fact that 95 per cent of the population is Yoruba. Of the 60 patients whose home address was known, 28 were from Ilesha township and 32 from surrounding villages. In assessing the home address the place mainly inhabited during the previous year was recorded. Those recording addresses have recently had to guard against a tendency for patients to use the Ilesha address where patients only live while attending hospital. It is impossible to assess the influence of this tendency in early years records, thus inflating the Ilesha numbers at the expense of the non-Ilesha. In the period 1954–61 inclusive there were 11 recorded cases from Ilesha and 4 from outside. In the last 5 years the balance has altered; 21 lived outside Ilesha compared to 9 in the township.

During the period under survey 50 other childhood tumours were seen. In the period 1954–61 inclusive 12 of these were from Ilesha township and 6 from outside. During the last 5 years there were 11 from Ilesha and 13 from outside.

(v) *Population structure.* In detailed population surveys in the Ijesha village of Imesi-Ile 25 miles north-east by road from Ilesha, Woodland (1966, personal communication) found the distribution of population by age as recorded in Table III. (Two previous surveys in 1960 and 1963 gave similar results.) For comparison figures from a W.H.O. survey in Ibadan, quoted by Edington and Maclean (1965) and the Arbitrary Standard Population for African Races (Knowelden and Oettlé, 1962) are given.

TABLE III.—*Percentage Distribution of Population by Age. (Total in Each Sex is* 100%)

| Age (years) | Imesi-Ile (1966) Male | Imesi-Ile (1966) Female | Ibadan (1962) Male | Ibadan (1962) Female | Arbitrary standard Male | Arbitrary standard Female |
|---|---|---|---|---|---|---|
| 0–4 | 20·9 | 16·4 | 16·7 | 16·6 | 10·0 | 10·0 |
| 5–9 | 20·8 | 14·8 | 12·2 | 14·1 | 10·0 | 10·0 |
| 10–14 | 14·0 | 10·7 | 10·0 | 9·6 | 10·0 | 10·0 |
| 15–19 | 8·4 | 5·0 | 9·4 | 6·3 | 10·0 | 10·0 |

With local population figures showing approximately 60 per cent of the population under 20 years, it follows that childhood tumours assume a much higher proportion of malignancies seen in a Nigerian general hospital by comparison with industrial society where the older population is at greater risk. Seventeen per cent of all tumour patients seen in Ilesha are under the age of 20 years. As almost half of these have Burkitt lymphoma this tumour represents 8·6 per cent of all cancers seen in Ilesha.

TABLE IV.—*Annual Age Specific Incidence in Burkitt's Lymphoma at Ilesha* (1964–69)

| | 0–4 | 5–9 | 10–14 |
|---|---|---|---|
| Numbers of patients seen in 6 years | 3 | 21 | 6 |
| Percentage of total population | 18·7 | 17·8 | 12·3 |
| Estimated population at risk per annum | 84,150 | 80,100 | 55,350 |
| Incidence/100,000/year | 0·6 | 4·4 | 1·8 |

(vi) *Tumour incidence* (Table IV). An attempt has been made to calculate the age specific rates using the Imesi statistics to estimate the numbers at risk in each age group. It is assumed for the purposes of calculation that the total Ijesha population remained static at 450,000 (1963 Census) during the period 1964–69, when it is felt, the numbers diagnosed most truly represent the actual incidence in the area. Of the total 32 patients from Ijesha divisions 30 were under 15 years old. Four of the 5 patients whose addresses are unknown are included in this figure on the assumption that, as 83 per cent of all cases of known address originate from the division, it is legitimate to assume the same proportions in those of unknown origin.

These figures are comparable to the previous report from Ilesha when figures of 2·4, 6·7 and 1·9 respectively for the three age groups were obtained from Ilesha township (Mulligan, 1970). The Standard Population for Africa (Knoweldon and Oettlé, 1962) was then used to estimate the local population. As reference to Table III reveals, this arbitrary standard under-estimates the younger age groups in this area and so the former figures are slightly exaggerated. It does however seem that the incidence in the Ilesha area is consistently and significantly less than in the Ibadan township which has figures of 1·7, 15·3 and 12·8 respectively for each 5-year period (Edington and Maclean, 1964).

Burkitt and Wright (1966) recorded the number of cases of this tumour seen per 100,000 total population over an 8-year period in different parts of Uganda. For comparison with their figures and using the 32 cases seen in Ijesha land from 1964 to 1969 the figure is 9·5. This approaches the maximum incidence (13·4) found in Uganda.

*Tumour geography*

The figure shows the distribution of tumours from outside Ilesha in relation to the main land contours. The area may be divided into two main sections. A ridge reaching up to 2200 feet to the east and rocky outcrops to the south of Ilesha contrast with gently undulating ground, never more than 100 feet above or below the 1200 feet contour, to the north-west and south-west of the town. Throughout this area there are slow flowing streams with dense undergrowth and profuse vegetation. The hillsides are also covered with trees. Apart from two villages at 1600 feet the majority of villages from which tumours came were near the 1200 feet contour.

Rainfall variation within the area is not accurately known. It falls mainly from May to October. The Ilesha annual average is 60 inches.

The dates on which different cases have presented from the same village or villages close together have been mapped. There is no evidence of any cluster effect or drift phenomenon as found by Williams (1966) and Pike *et al.* (1967) though the statistical techniques used by the latter were not applied. Two patients from Effon Alaye presented within 10 weeks of each other, one with an abdominal mass and the other with a maxillary tumour. From the addresses given it was not possible to work out the distance separating the children within the village, though it should be noted that the village home is often only a lodging centre, far from the farm where a large part of the week is spent.

### DISCUSSION

This survey has confirmed the now classical pattern of presentation described by Burkitt (1966). As O'Conor and Davies (1960) first stressed, approximately one half of the patients have facial involvement. As they also observed, half of the patients with facial lesions have no other gross pathology. This is confirmed here in 12 out of 25 patients. The male to female ratio, as found by others, is inexplicably 3 : 1 for facial tumour but 1 : 1 for other sites. This have never been adequately explained, though the preponderance of head over trunk lesions in the younger age groups, also confirmed here, may be related to the almost continuous coverage of the whole child on the mother's back while only the face and neck are exposed to attack by insect vectors.

The finding of Burkitt and Wright (1966) that facial tumours develop in young adults lately arrived in the tumour endemic area shows that the facial localization as once postulated is not related to rapid dental activity in the 2- and 3-year old.

Burkitt and Wright (1966) have shown that the age of patients is highest in areas where the incidence is lowest though Pike *et al.* (1967) found the opposite tendency. Consideration of the age scatter in Ilesha as seen in Table II, with only 3 cases over the age of 14 years, suggests that this district is in the centre of a tumour area, which is known to extend West beyond Ibadan, 75 miles away. In Ibadan the age scatter was 2–27 years (Edington and Maclean, 1964). The comparative relative ratio frequencies for tumours in all age groups were 8·9 in Ibadan and 8·6 in Ilesha (Mulligan, 1970). The only other tumour surveys from Nigeria with specific reference to Burkitt lymphoma were from Lagos (Duncan, 1968), with a relative ratio of 3·4, and the Northern States Pathology reference laboratory (Berry, 1964) with a relative ratio of 5·7. Berry stated that most of his biopsies showing Burkitt's lymphoma were from the Niger/Benue river basin. From discussions with doctors working near the Benue river one has the impression that it may yet prove to have one of the highest concentrations of this tumour in Nigeria.

By relating the numbers seen in a fixed population over an 8-year period an attempt has been made to relate experience with that in the well documented high tumour density West Nile district of Uganda though relative ratios for childhood tumours based on total population surveys must be interpreted against the background of the population age scatter. The estimated number of cases per 100,000 total population over a fixed period could be deceptive without details of population age distribution. Nevertheless the calculated ratio 9·5 : 13·4 does put local findings within a wider context. On the other hand Ibadan township has, in terms of the crude age specific rates, an incidence three times that of Ilesha. It therefore appears that the Western half of the West State is an important high density tumour area, probably among the most dense in Africa.

During the last few years there has been a significantly reduced proportion of patients with Burkitt's lymphoma coming from the Ilesha township. No cases during the whole survey period have come from Imesi-Ile with a population of 6000. This village, which has been the focus of concentrated medical research since 1957, is one of the healthiest villages in West Africa (Morley, 1963). Any tumours would certainly have been noted by the resident health sister. On the basis of experience in the whole area under survey at least 1 and possibly 2 cases would have been expected during the 16 years. In both Imesi and Ilesha at least 95 per cent of the children hold cards for the respective welfare clinics where preventive inoculations are given and fevers treated with anti-malarials—without charge. Children in Imesi have had monthly Daraprim. By contrast other outlying villages, especially those along the base of the eastern ridge of hills having scanty preventive and curative facilities, are now the main source of Burkitt's lymphoma. In the past such patients may not have been seen at hospital due to poor transport facilities and continued belief in traditional remedies and healers. In the peripheral villages a specific increase in cases of Burkitt's lymphoma compared with other childhood tumours has been noted.

The association of Burkitt's lymphoma and malaria was initially suggested by

O'Conor (1961), but only recently has the relationship between holo-endemic malaria and the tumour been convincingly demonstrated (Burkitt, 1969; Kafuko *et al.*, 1969; O'Conor, 1970; *Lancet*, 1970). It is now conceived that the reticulo-endothelial system overstimulated by malaria is a fertile substrate for an oncogenic virus, probably that causing infectious mononucleosis (Henle and Henle, 1969). In Imesi and Ilesha where anti-malarials, both prophylactic and therapeutic, are freely available it has been shown that these children are slower than controls to develop gamma-globulins—due, presumably, to less intense stimulation of the reticuloendothelial system (Edozien *et al.*, 1960). There were 11 cases seen in Ilesha before 1962; only 4 were 8 years or more. Since 1962, 10 out of 17 were over 8 years old. By contrast only 10 in 28 non-Ilesha patients since 1962 were 8 years old or over. The age pattern in all other childhood neoplasms has not altered. Burkitt (1969) quotes several authors to show the inverse relationship between malaria eradication and the density of Burkitt's tumour. The experience in Ilesha demonstrates an earlier stage in the same process, namely, delay in age of onset due to reduced malarial stimulation.

Another factor may operate. The high level of immunity produced by B.C.G., triple vaccine, polio and small-pox vaccines alerts not only the defense mechanisms of the body against these specific infections but also generally against possible oncogenic viruses or virus co-carcinogens. There is also a non-specific immuno-therapeutic effect of vaccination upon tumours which, though weak in itself, may assist other factors in delaying or resisting tumour development (Mathé, 1967; Fairley, 1969). It would, in addition, have been interesting to have records of the red cell sickling status of each patient, but this was not routinely carried out, even though facilities for doing so have been available.

My thanks are due to Professor O. A. Williams of Ibadan University for initial helpful suggestions; to Mrs Hendrickse and staff of the Ibadan Tumour Registry for tracing all cases of Burkitt's lymphoma registered from the Ilesha area; to Mr. Denis Burkitt and, especially, Miss Paula Cook of the M.R.C. External Scientific Staff, London, who has helped with the figure and advised on the statistical method.

This paper was presented at the Second International Cancer Conference, Lagos, Nigeria, 17–19 December, 1970.

### REFERENCES

Berry, C. G.—(1964) *Br. med. J.*, ii, 668.
Burkitt, D.—(1958) *Br. J. Surg.*, **46**, 218.—(1962) *Br. J. Cancer*, **16**, 379.—(1966) *Jl R. Coll. Surg. Edinb.*, **11**, 170.—(1969) *J. natn. Cancer Inst.*, **42**, 19.
Burkitt, D. and Wright, D. H.—(1966) *Br. med. J.*, i, 569.
Duncan, J. T. K.—(1968) *W. Afr. med. J.*, **17**, 96.
Edington, G. M. and Maclean, C. M. H.—(1964) *Br. med. J.*, i, 264.—(1965) *Br. J. Cancer*, **19**, 470.
Edozien, J. C., Boyo, A. E. and Morley, D. C.—(1960) *J. clin. Path.*, **13**, 118.
Eshlemann, J. L.—(1966) *E. Afr. med. J.*, **43**, 273.
Fairley, G. H.—(1969) *Br. med. J.*, ii, 464.
Henle, W. and Henle, G.—(1969) *E. Afr. med. J.*, **46**, 402.
Hutt, M. R. S. and Burkitt, D.—(1965) *Br. med. J.*, ii, 719.
Kafuko, G. W., Baingana, N., Knight, E. M. and Tibemanya, J.—(1969) *E. Afr. med. J.*, **46**, 414.

KNOWELDON, J. AND OETTLÉ, A. G.—(1962) Unpublished data used by Davies, J. N. P., Wilson, B. A. and Knoweldon, J. (1962) *Lancet*, ii, 328.
*Lancet*—(1970) ii, 300.
MATHÉ, G.—(1967) *Ann. R. Coll. Surg.*, **41**, 93.
MORLEY, D. C.—(1963) *Trans. R. Soc. trop. Med. Hyg.*, **57**, 79.
MULLIGAN, T. O.—(1970) *Br. J. Cancer*, **24**, 1.
O'CONOR, G. T.—(1970) *Am. J. Med.*, **48**, 279.—(1961) *Cancer, N.Y.*, **14**, 270.
O'CONOR, G. T. AND DAVIES, J. N. P.—(1960) *Pediatrics, Springfield*, **56**, 526.
PIKE, M., WILLIAMS, E. H. AND WRIGHT, B.—(1967) *Br. med. J.*, ii, 395.
WILLIAMS, E. H.—(1966) *E. Afr. med. J.*, **43**, 200.